# Neurexin-Neuroligin Synaptic Complex Regulates Schizophrenia-Related DISC1/Kal-7/Rac1 “Signalosome”

**DOI:** 10.1155/2015/167308

**Published:** 2015-05-20

**Authors:** Sylwia Owczarek, Marie Louise Bang, Vladimir Berezin

**Affiliations:** ^1^Laboratory of Neural Plasticity, Department of Neuroscience and Pharmacology, University of Copenhagen, 2200 Copenhagen, Denmark; ^2^Research Laboratory for Stereology and Neuroscience, Bispebjerg Hospital, 2400 Copenhagen, Denmark

## Abstract

Neurexins (NXs) and neuroligins (NLs) are cell adhesion molecules that are localized at opposite sites of synaptic membranes. They interact with each other to promote the assembly, maintenance, and function of synapses in the central nervous system. Both NX and NL are cleaved from a membrane-attached intracellular domain in an activity-dependent manner, generating the soluble ectodomain of NX or NL. Expression of the *NX1* and *NX3* genes in the brain appears to be regulated by a schizophrenia-related protein, DISC1. Here, we show that soluble ecto-NX1*β* can regulate the expression of DISC1 and induce signaling downstream of DISC1. We also show that NL1 binds to a well-characterized DISC1 interaction partner, Kal-7, and this interaction can be compromised by DISC1. Our results indicate that the NX/NL synaptic complex is intrinsically involved in the regulation of DISC1 function, thus contributing to a better understanding of the pathology of schizophrenia.

## 1. Introduction

The development and maintenance of synaptic connections are a dynamic process that requires bidirectional interactions between pre- and postsynaptic components. Adhesion molecules are present at the synapse and organize synaptic specializations. The synaptic adhesion complex neurexin/neuroligin (NX/NL) mediates synapse formation and triggers pre- and postsynaptic signal transduction by recruiting components to developing synapses [1]. Depending on the composition of NX/NL complexes, they function as molecular switches that regulate excitatory-inhibitory (E/I) balance in the brain [1]. Mammals have three* NX* genes, each with two promoters. Each gene produces both longer *α*-NX and shorter *β*-NX transcripts. Additionally, each isoform can be alternatively spliced at five and two positions, respectively [1]. Humans express five* NL* genes, whereas other mammals express only three or four [1]. Each* NL* gene has a canonical splicing site (SS#A), and NL1 additionally has the SS#B splicing site. Interestingly, the expression and function of both NXs and NLs are regulated by neural activity.* NX* mRNA expression increases as synaptic activity increases [2], and both NX and NL can be cleaved from the pre- or postsynaptic membrane in an activity-dependent manner [3–6]. The expression of the* NX1* and* NX3* genes was recently shown to be dysregulated in the* Disc1* (disrupted in schizophrenia 1) mouse model of schizophrenia [7]. DISC1 is a scaffolding protein that plays several roles during many aspects of neural development [8]. For example, the inactivation of DISC1 during early development results in alterations in the density and morphology of dendritic spines [9]. Spines appear to be regulated by DISC1 in association with kalirin-7 (Kal-7) [10–12]. Kal-7 is an activator of a small guanosine triphosphatase (GTP), Rac1. DISC1, together with Kal-7 and Rac1, forms a multifunctional protein complex, called the DISC1/Kal-7/Rac1 signalosome, that is important for development of the nervous system [10]. Under baseline conditions, DISC1 binds Kal-7, blocking the access of Kal-7 to Rac1. The activation of ionotropic glutamate receptors (*N*-methyl-D-aspartate [NMDA] receptors) weakens this protein-protein interaction, allowing free access of Kal-7 to Rac1, which leads to the activation of Rac1 and eventually spine maturation [10]. The aforementioned findings led us to hypothesize that NX/NL- and DISC1-induced signaling might be interconnected. Here, we show that NL1 binds to Kal-7, and alterations in NX-NL interactions lead to disturbances in the DISC1/Kal-7/Rac1 signalosome, thus allowing the activation of Rac1.

## 2. Materials and Methods

### 2.1. Materials

The recombinant ectodomain of human neurexin 1 beta Fc Chimera (ecto-NX1*β*) was obtained from R&D Systems (catalog number 5268-NX-050; Abingdon, UK). An expression vector that encodes NL1-AB, pCAGNL1-AB was a gift from Peter Scheiffele (Addgene plasmid #15262) [13]. An expression vector that encodes Kal-7, pEAK10-His-Myc-Kal7 was a gift from Betty Eipper (Addgene plasmid #25454) [14]. An expression vector that encodes mouse FLAG-myc-tagged DISC1 was purchased from Origene (Rockville, MD, USA). The PKA inhibitor H89 was obtained from Invivogen (Toulouse, France). The following antibodies were used in this study: rabbit anti-S897-NR1, anti-S896-NR1 (1 : 500; Millipore, Billerica, MA, USA), phospho-Erk1/2, phospho-Akt (1 : 1000; Cell Signaling, Beverly, MA, USA), mouse anti-*β*-tubulin (1 : 50,000; Sigma-Aldrich, St. Louis, MO, USA), anti-HA tag (Abcam, Cambridge, UK), anti-myc-tag (Sigma-Aldrich), anti-Kal-7 (Abcam), and IRDye secondary antibodies (1 : 20,000; Odyssey, Lincoln, NE, USA).

### 2.2. Cell Cultures

Cortical neurons were obtained on embryonic day 18 from Wistar rat embryos (Charles River Laboratories, Kisslegg, Germany) as described previously [15]. For each single experiment, the cortices from all embryos were dissected, cleared from blood vessels and meninges, pooled together, mechanically chopped, and trypsinized. The cells were then washed in the presence of DNase and trypsin inhibitor, resuspended, and seeded at a density of 2 × 10^5^ cells/cm^2^ in poly-D-lysine coated (1 *μ*g/mL) 6 cm Petri dishes (Nunc, Roskilde, Denmark) in Neurobasal medium supplemented with 2% (v/v) B27, 1% (v/v) GlutaMax, 100 U/mL penicillin, 100 *μ*g/mL streptomycin, and 2% (v/v) of 1 M HEPES (all from Gibco, Waltham, MA, USA). At 3 DIV, cytosine *β*-D-arabinofuranoside (Ara-C) in concentration 2 *μ*M was added to inhibit the growth of nonneuronal cells. Cortical neurons were grown for 8 days. At this developmental stage the majority of synapses are immature. The advantage of using these young cultures is the high survival rate of neurons and general low background due to low level of spontaneous activity [16].

Human embryonic kidney 293 (HEK293) cells were obtained from the European Cell Culture Collection (Salisbury, UK) and maintained in Dulbecco's Modified Eagle's Medium (DMEM) supplemented with 10% (v/v) fetal calf serum, 2 mm GlutaMax, 100 units/mL penicillin, and 100 *μ*g/mL streptomycin. All of the cell cultures were kept in a humidified incubator at 37°C in a 5% CO_2_ atmosphere.

### 2.3. Immunoblotting

Cortical neurons were grown for 8 days before treatment. Thereafter, neurons were stimulated with ecto-NX1*β* (0.01, 0.05, and 0.2 nM) for 5 min. The cells were lysed, and proteins were separated by sodium dodecyl sulfate-polyacrylamide gel electrophoresis (SDS-PAGE) followed by immunoblotting using the antibody of interest. The bands were visualized using the Odyssey CLX Infrared Imaging System (Odyssey).

### 2.4. Rac1-GTP Assay

Cortical neurons were grown for 8 days and stimulated with ecto-NX1*β* (0.01, 0.05, and 0.2 nM) for 20 min. The activation of Rac1 was measured with the Rac1 activity assay kit (Thermo Scientific, Rockford, IL, USA) according to the manufacturer's instructions. A fusion protein that consisted of the p21-binding domain of Pak1, which specifically binds to the active form of Rac1 (Rac-GTP) but not to the inactive form of Rac1 (Rac-GDP), was used for Rac-GTP precipitation. Immunoprecipitated samples were analyzed by SDS-PAGE and immunoblotting using a primary mouse anti-Rac1 antibody (1 : 1000; Thermo Scientific) and goat anti-mouse RDye secondary antibodies (1 : 20,000; Odyssey).

### 2.5. Real-Time Quantitative Polymerase Chain Reaction

Cortical neurons were grown for 8 days and treated with ecto-NX1*β* (0.01, 0.05, and 0.2 nM) for 2 h (c-*fos*), 24 h (DISC1), or 48 h (NR1 splice variants). Total RNA was prepared using the GeneJET RNA Purification Kit (Thermo Scientific) according to the manufacturer's instructions. RNA integrity and quantity were verified using an Agilent 2100 Bioanalyzer (Agilent Technologies). Only samples with RIN ≥ 8 were used for further analyses. The RNA samples were stored at −80°C until use. Quantitative reverse-transcription polymerase chain reaction (PCR) and the data analyses were performed as described previously [17] using the primers that are shown in Table 1. All of the samples were run in duplicate. The relative levels of the PCR products in all of the samples were evaluated by the Pfaffl method [18].

### 2.6. Coimmunoprecipitation

HEK293 cells were transfected with cDNA expression vectors that encoded the proteins of interest. Forty-eight hours after transfection, cells were lysed in immunoprecipitation (IP) buffer (phosphate-buffered saline [PBS] and 1% NP40) supplemented with complete protease inhibitors (Roche), and cleared lysates were incubated with appropriate antibodies overnight. Immunoprecipitates were collected with Pierce protein A/G magnetic beads (Thermo Scientific), washed twice in IP buffer (once with PBS),and analyzed by SDS-PAGE and immunoblotting.

### 2.7. Data Analysis

The statistical analysis was performed using one-way analysis of variance (ANOVA) followed by Dunnett's multiple-comparison* post hoc* test, Sidak's multiple-comparison* post hoc* test, or one-sample* t*-test when appropriate using Prism 6 software (GraphPad, San Diego, CA, USA).

## 3. Results and Discussion

**Figure 1:**
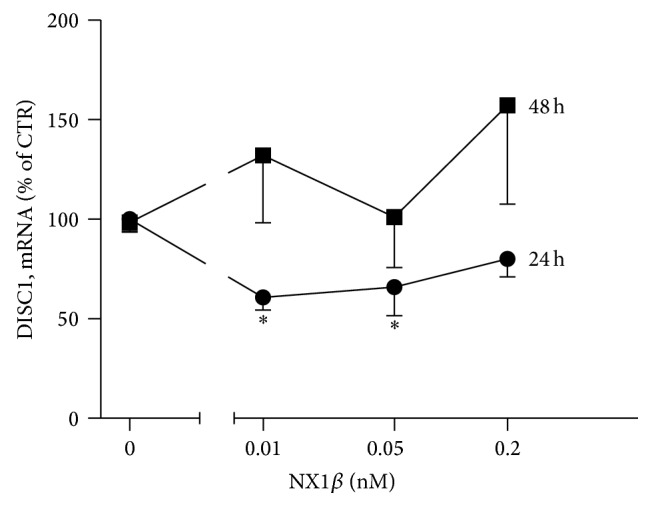
NX1*β* decreases the mRNA level of DISC1. Cortical neurons were grown for 8 days and subsequently treated with different concentrations of recombinant NX1*β* for (a) 24 h and (b) 48 h. Total RNA was isolated, and mRNA levels were assessed using RT-PCR. The results from *n* = 3 experiments are expressed as a percentage ± SEM. ^*∗*^ *P* < 0.05, compared with the untreated control set at 100%.

The expression of the* NX1* gene appears to be regulated by a DISC1-dependent mechanism [7], and this regulation is important for brain development and function. We investigated whether this regulation might be reciprocal and whether NX might also affect the expression of* Disc1*. Indeed, we found that 24 h treatment with ecto-NX1*β* decreased the mRNA level of* Disc1 *(Figure 1). This effect was inversely proportional to the dose of the protein. The highest concentration had no effect on* Disc1* expression. Notably, the longer 48 h treatment with ecto-NX1*β* did not have a significant effect on* Disc1* expression (Figure 1).

**Figure 2:**
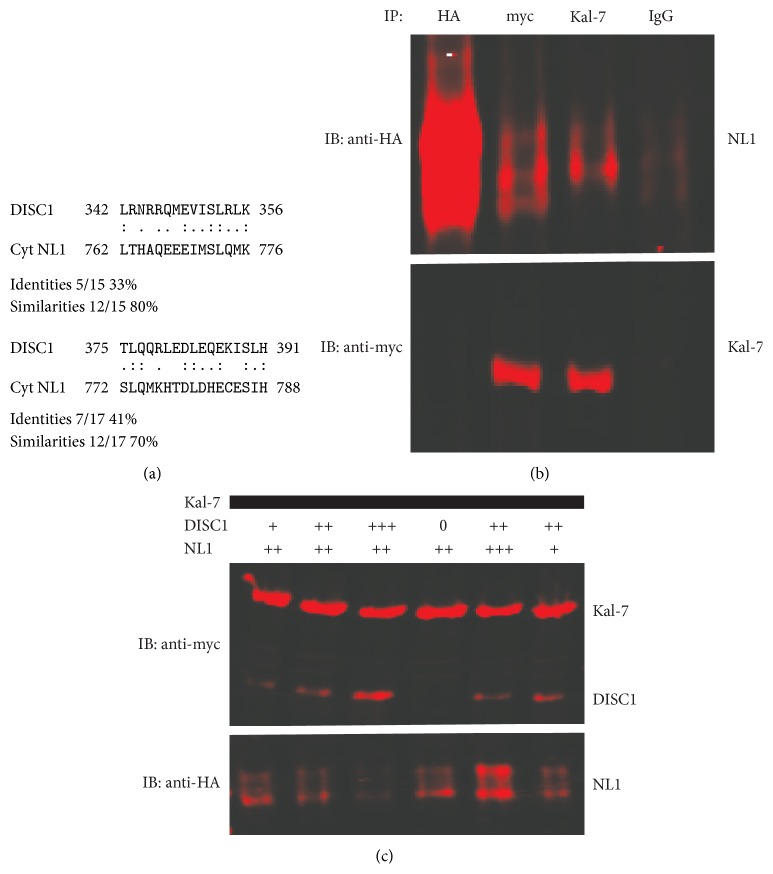
Neuroligin 1 interacts with kalirin 7. (a) 293T cells were transfected with HA-tagged NL1-AB or myc-tagged Kal-7. Cell lysates were mixed and subsequently immunoprecipitated with anti-HA, anti-myc, or anti-Kal antibody, and immunoblots were probed with the indicated antibodies. Mouse IgG was used as a control. (b) HEK293 cells were transfected with HA-tagged NL1-AB or myc-tagged Kal-7 or FLAG-tagged DISC1. Cell lysates were mixed as indicated and subsequently immunoprecipitated. Immunoblots were probed with the indicated antibodies. Representative immunoblots of *n* = 3 experiments are shown.

DISC1 has many identified protein interaction partners, including synaptic proteins [8]. We hypothesized that either NX or NL might interact with DISC1 itself or might affect DISC1 interactions with other partners. Using LALIGN [18] to find the best local alignments between two sequences, we inspected the cytoplasmic domains of both NX1 (EAX00186.1) and NL1 (ADB12633.1) for homology to DISC1 (Q9NRI5.3). We found that the cytoplasmic region of NL1 contains at least two sequence motifs that are homologous to DISC1 (Figure 2(a)). Moreover, these motifs are located in the DISC1 domain, which is crucial for binding with Kal-7 (amino acids 350–394 in DISC1) [10]. Hence, we examined whether NL1 might directly interact with Kal-7. The interaction was confirmed by co-IP from heterologous cells (Figure 2(b)). Moreover, in the presence of DISC1, the binding of Kal-7 to NL1 decreased substantially (Figure 2(c)), suggesting possible competition between DISC1 and NL1 for binding to Kal-7. In contrast, the presence of NL1 did not affect the DISC1-Kal-7 interaction, suggesting that DISC1 must first be released from Kal-7 to allow NL1-Kal-7 interactions.

**Figure 3:**
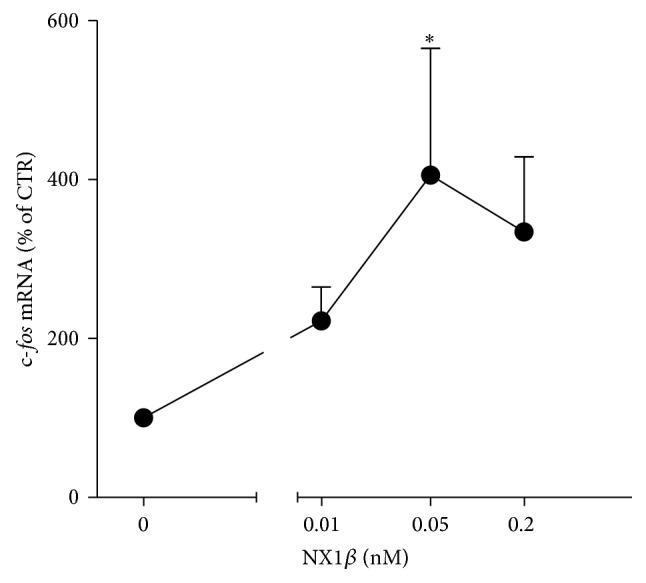
NX1*β* activates Rac1. Cortical neurons were grown for 8 days and subsequently treated with different concentrations of recombinant NX1*β* for 20 min, and the level of active Rac1 (Rac1-GTP) was analyzed using immunoblotting. The results from *n* = 4 experiments are expressed as a percentage ± SEM. ^*∗*^ *P* < 0.05, compared with the untreated control set at 100%.

**Figure 4:**
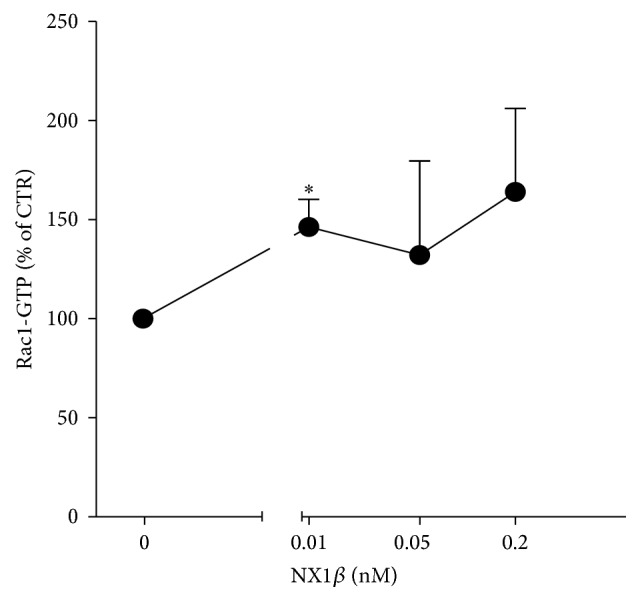
NX1*β* increases the mRNA level of the immediate early gene c-*fos*. Cortical neurons were grown for 8 days and treated with different concentrations of recombinant NRXN1b for 2 h. Total RNA was isolated, and mRNA levels were assessed using RT-PCR. The results from *n* = 4 experiments are expressed as a percentage ± SEM. ^*∗*^ *P* < 0.05, compared with the untreated control set at 100%.

The interaction between Kal-7 and DISC1 depends on synaptic activity. Upon neuronal activation, DISC1 dissociates from Kal-7, which in turn leads to the activation of Rac1 [10]. Neuronal activity also leads to the destabilization and shedding of both NX1 and NL1 from the synaptic membrane [3–6]. Additionally, treatment with recombinant ecto-NX1*β* mimics some of the effects of neuronal activity associated with the cleavage of NL1 from the postsynaptic membrane [6]. We observed that treatment with ecto-NX1*β* resulted in an increase in the mRNA level of the immediate early gene c-*fos* (Figure 3), indirectly supporting the hypothesis that treatment with soluble NX1 augments neuronal activity. Thus, we tested whether ecto-NX1*β* itself affects Rac1 activation. As shown in Figure 4, ecto-NX1*β* increased the level of Rac1-GTP at a concentration as low as 0.01 nM. At higher concentrations, ecto-NX1*β* did not have a statistically significant effect on Rac1-GTP.

**Figure 5:**
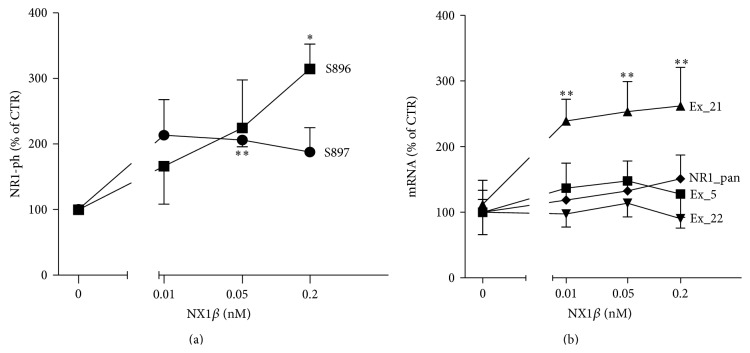
NX1*β* differentially affects the phosphorylation and expression of NR1. (a) Cortical neurons were grown for 8 days, subsequently treated with different concentrations of recombinant NX1*β* for 5 min, and further immunoblotted for the detection of NR1 phosphorylation at S896 and S897. Representative immunoblots of three to four individual experiments are shown. The results from *n* ≥ 4 experiments are expressed as a percentage ± SEM. ^*∗*^ *P* < 0.05, compared with the untreated control set at 100%. (b) Cortical neurons were grown for 8 days and treated with different concentrations of recombinant NX1*β* for 48 h. Total RNA was isolated, and mRNA levels were assessed using RT-PCR. The results from *n* = 5 experiments are expressed as a percentage ± SEM. ^*∗*^ *P* < 0.05, compared with the untreated control set at 100%.

The signaling of Kal-7 to Rac1 is coupled with neuronal activity via NMDA receptors [10]. Therefore, we examined whether NX can modulate the phosphorylation levels of an obligatory subunit of the NMDA receptor, NR1. NR1 can be phosphorylated at three distinct serine residues (S897, S896, and S890) in the intracellular carboxyl tail region [19]. Active protein kinase C (PKC) phosphorylates the NR1 subunit at S890 and S896 [20], whereas the activation of cyclic adenosine monophosphate- (cAMP-) dependent protein kinase A (PKA) leads to phosphorylation at S897 [21]. As shown in Figure 5(a), treatment with ecto-NX1*β* led to phosphorylation at both S896 and S897. Interestingly, the level of Ser896 was markedly increased by the highest concentration of ecto-NX1*β* (0.2 nM), whereas the level of Ser897 was significantly increased by 0.05 nM ecto-NX1*β*. In contrast to PKC, PKA responses are strong and brief. One of the targets that PKA phosphorylates and activates is phosphodiesterase, which rapidly lowers cAMP levels [22]. This might explain the differential effect of ecto-NX1*β* on PKA- and PKC-dependent phosphorylation sites in NR1.

NR1 exists in many isoforms through the alternative splicing of exons 5, 21, and 22. Exon 5, which encodes the extracellular N-terminal domain, is known to modulate the pharmacological properties of NMDA receptors [19]. Exon 21 and exon 22 (also known as the C1 and C2 cassettes, resp.) encode the intracellular C-terminal domain. mRNA splicing at these exons regulates protein-protein interactions, receptor trafficking, and NR1 phosphorylation [19]. We found that long-term 48 h treatment but not 24 h treatment (data not shown) with ecto-NX1*β* dose-dependently increased the relative expression levels of the* NR1-C1* splice variant (Figure 5(b)) but not other splice variants.

The C1 cassette contains two independent endoplasmic reticulum (ER) retention motifs and plays a unique role in the trafficking of NR1 subunits to the cell surface. Thus, the effect of ecto-NX1*β* on NMDA receptor trafficking appears to be time-dependent because short-term treatment increased NR1 phosphorylation, which might promote the forward trafficking of NMDA receptors to the surface, but prolonged treatment led to the retention of NR1 in the ER.

**Figure 6:**
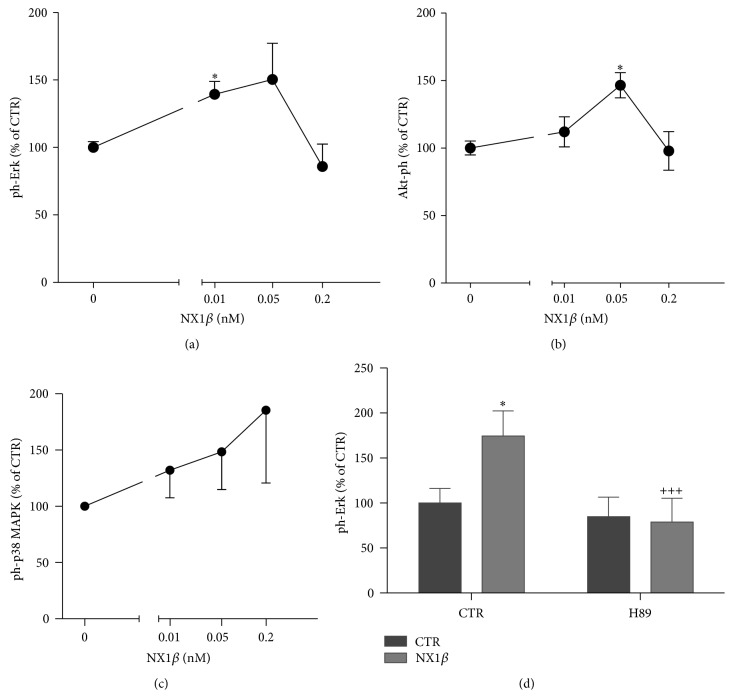
NX1*β* activates ERK1/2 and Akt kinases. Cortical neurons were grown for 8 days, subsequently treated with different concentrations of recombinant NX1*β* for 5 min, and further immunoblotted for the detection of (a) ERK1/2, (b) p38 MAPK, and (c) Akt. (d) Effect of the PKA inhibitor H89 on NX1*β*-induced ERK1/2 phosphorylation. The results from *n* ≥ 4 experiments are expressed as a percentage ± SEM. ^*∗*^ *P* < 0.05, compared with the untreated control set at 100%.

Finally, we investigated which signaling pathways are activated by ecto-NX1*β* downstream of the NMDA receptor and Kal-7. Three kinases that represent three different signaling cascades, ERK1/2, p38 MAPK, and Akt (PKB), are known to mediate signal transduction that underlies neuronal activity in response to the activation of NMDA receptors [23, 24]. As shown in Figure 6, ecto-NX1*β* had an effect on the phosphorylation levels of ERK1/2 (Figure 6(a)) and Akt (Figure 6(b)) but not p38 (Figure 6(c)). The effect on ERK1/2 and Akt was most pronounced at low concentrations and not seen at the highest concentration. To confirm the involvement of PKA in the NX-induced signaling pathway, we concomitantly treated cultured cortical neurons with ecto-NX1*β* and the specific PKA inhibitor H89. H89 abolished the effect of 10 *μ*M ecto-NX1*β* on ERK1/2 phosphorylation and had no effect in control (nonstimulated) cultures (Figure 6(d)). We did not observe any effect of the PKC inhibitor Calphostin C on NX-mediated ERK1/2 phosphorylation (data not shown).

**Figure 7:**
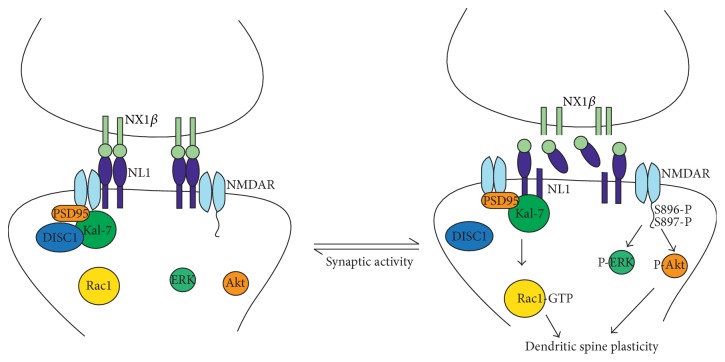
Schematic illustration of possible consequences of the NR1:NL1 interaction with the DISC1/Kal-7/Rac1 signalosome. At the baseline condition, NL1 forms homodimer interacting with NMDA receptor while DISC1 binds Kal-7, blocking the access of Kal-7 to Rac1. Upon synaptic activity and activation of NMDA receptors, NX1*β* is cleaved from the presynaptic membrane leading to partial cleavage of NL1 from the postsynaptic membrane followed by the release of NL1 from NMDA receptor and DISC1 from Kal-7. After the shedding, NL1 is no longer a dimer which probably changes the mobility of N-terminally truncated NL1, and it can diffuse allowing NL1 to bind Kal-7 and in turn lead to the activation of Rac1. These events eventually allow NL1 to bind Kal-7 and in turn lead to the activation of Rac1. Additionally, binding of ecto-NX1*β* t*ο* NL1 results in NR1 subunit phosphorylation at S896 and S897 and phosphorylation of ERK and Akt.

Our data indicate that the NX1:NL1 complex is involved in the formation of the DISC1/Kal-7/Rac1 “signalosome,” which is an important determinant of the neuronal activity-dependent morphological and functional plasticity of dendritic spines [10]. The overexpression of either Kal-7 [14] or Rac1 led to an increase in spine size [10], and a similar effect was observed with DISC1 downregulation [10]. The NX-NL complex is also involved in remodeling dendritic spines. Importantly, NL can act by itself to increase the number of synapses, but NX-NL binding is essential for the increase in apparent synapse size [25]. Except NX, NL1 also interacts extracellularly with NMDA receptor and this interaction is crucial for the synaptic recruitment and retention of NMDARs at glutamatergic synapses [26]. We suggest (see Figure 7) that, at the baseline condition, NL1 forms a homodimer [1] interacting with NMDA receptor while DISC1 binds Kal-7, blocking the access of Kal-7 to Rac1. Upon synaptic activity and activation of NMDA receptors, NX1*β* is cleaved from the presynaptic membrane what leads to the partial cleavage of NL1 from the postsynaptic membrane followed by the release of NL1 from NMDA receptor and DISC1 from Kal-7. After the shedding, NL1 is no longer a dimer which probably changes the mobility of N-terminally truncated NL1, and it can diffuse allowing NL1 to bind Kal-7 and in turn lead to the activation of Rac1. Additionally, binding of ecto-NX1*β* t*ο* NL1 results in NR1 subunit phosphorylation at S896 and S897 and phosphorylation of ERK and Akt. Interestingly, the 24 h exposure to ecto-NX1*β* results in the diminished level of* DISC1* expression (Figure 1) and increased NL1 shedding from the cell surface [6] while prolonged (48 h) treatment with ecto-NX1*β* did not have any effect on mRNA level of* DISC1* suggesting that all these events are reversible, in a sort of equilibrium, because the dynamics of these events determines spine plasticity.

Some mutations and structural variations in both the* NX1* and* DISC1* genes have been associated with schizophrenia and autism spectrum disorders [26–28]. The common denominator for these diseases is most likely disturbances in neuronal connectivity in the brain [29, 30]. Interestingly, the abnormal expression of* NX1 *in mice with mutated* DISC1* is most prominent during the stages of synapse formation, neuronal maturation, and the general development of neuronal connectivity [7]. The DISC1/Kal-7/Rac1 signalosome also plays a role in neuronal development and may also contribute to the juvenile onset of mental diseases [31–33]. Our data support the functional connection between the DISC1/Kal-7/Rac1 signalosome and NX1*β*-NL1 complex. This interaction might play an important role in establishing proper neural connectivity, and any disturbance of which might underlie pathologies seen in schizophrenia and autism spectrum disorders.

## 4. Conclusion

We found physical and functional connections between the NX1*β*-NL1 complex and the DISC1/Kal-7/Rac1 signalosome. The NX1*β*-NL1 complex appears to regulate the signalosome, and this regulation is most likely closely coupled with synaptic activity and the activation of NMDA receptors.

## Figures and Tables

**Table 1:** List of primers used in the study.

Gene	Accession number	Primer		Fragment size (bp)
		Sense	Antisense	
*c-fos *	NM_022197.2	5′ GGA GGA GGG AGC TGA CAG ATA C 3′	5′ GGT CAT TGG GGA TCT TGC AGG 3′	159
*Disc1 *	NM_175596.2	5′ TGC ACT CTG GGG TTC ATT CCG 3′	5′ CCA TTC GAC GCC GGT GAT 3′	190
*NR1-N1 *	L08228.1	5′ ATC ATC CTG CTG GTC AGC GAC G 3′	5′ GCA CCT TCT CTG CCT TGG GTC C 3′	160
*NR1-C1 *	L08228.1	5′ CGT GAA CGT GTG GAG GAA GAA C 3′	5′ CTA CGT CTC TTG AAG CTG GAG G 3′	121
*NR1-C2 *	L08228.1	5′ CTC AAC CTC TCA GAT CCC TC 3′	5′ GCA GCA GGA CTC ATC AGT GT 3′	102
*NR1-pan *	L08228.1	5′ TGT ACG TCA AGC CCA CAA TGA G 3′	5′ ACC AGG TGC ACC TCA TAG GTA 3′	211
*GAPDH *	NM_017008.3	5′ AGC TGG TCA TCA ACG GGA AAC C 3′	5′ CCT TCT CCA TGG TGG TGA AGA C 3′	126

## References

[1] Bang M. L., Owczarek S. (2013). A matter of balance: Role of neurexin and neuroligin at the synapse. *Neurochemical Research*.

[2] Kitamura C., Takahashi M., Kondoh Y., Tashiro H., Tashiro T. (2007). Identification of synaptic activity-dependent genes by exposure of cultured cortical neurons to tetrodotoxin followed by its withdrawal. *Journal of Neuroscience Research*.

[3] Bot N., Schweizer C., Halima S. B., Fraering P. C. (2011). Processing of the synaptic cell adhesion molecule neurexin-3*β* by Alzheimer disease *α*- and *γ*-secretases. *Journal of Biological Chemistry*.

[4] Peixoto R. T., Kunz P. A., Kwon H. (2012). Transsynaptic signaling by activity-dependent cleavage of neuroligin-1. *Neuron*.

[5] Saura C. A., Servián-Morilla E., Scholl F. G. (2011). Presenilin/*γ*-secretase regulates neurexin processing at synapses. *PLoS ONE*.

[6] Suzuki K., Hayashi Y., Nakahara S. (2012). Activity-dependent proteolytic cleavage of neuroligin-1. *Neuron*.

[7] Brown S. M., Clapcote S. J., Millar J. K. (2011). Synaptic modulators *Nrxn1* and *Nrxn3* are disregulated in a *Disc1* mouse model of schizophrenia. *Molecular Psychiatry*.

[8] Bradshaw N. J., Porteous D. J. (2012). DISC1-binding proteins in neural development, signalling and schizophrenia. *Neuropharmacology*.

[9] Lee F. H. F., Fadel M. P., Preston-Maher K. (2011). *Disc1* point mutations in mice affect development of the cerebral cortex. *Journal of Neuroscience*.

[10] Hayashi-Takagi A., Takaki M., Graziane N. (2010). Disrupted-in-Schizophrenia 1 (DISC1) regulates spines of the glutamate synapse via Rac1. *Nature Neuroscience*.

[11] Millar J. K., Christie S., Porteous D. J. (2003). Yeast two-hybrid screens implicate DISC1 in brain development and function. *Biochemical and Biophysical Research Communications*.

[12] Xie Z., Srivastava D. P., Photowala H. (2007). Kalirin-7 controls activity-dependent structural and functional plasticity of dendritic spines. *Neuron*.

[13] Chih B., Gollan L., Scheiffele P. (2006). Alternative splicing controls selective trans-synaptic interactions of the neuroligin-neurexin complex. *Neuron*.

[14] Penzes P., Johnson R. C., Alam M. R., Kambampati V., Mains R. E., Eipper B. A. (2000). An isoform of Kalirin, a brain-specific GDP/GTP exchange factor, is enriched in the postsynaptic density fraction. *The Journal of Biological Chemistry*.

[15] Ditlevsen D. K., Owczarek S., Berezin V., Bock E. (2008). Relative role of upstream regulators of Akt, ERK and CREB in NCAM- and FGF2-mediated signalling. *Neurochemistry International*.

[16] Lin Y.-C., Huang Z.-H., Jan I. S. (2002). Development of excitatory synapses in cultured neurons dissociated from the cortices of rat embryos and rat pups at birth. *Journal of Neuroscience Research*.

[17] Brudek T., Winge K., Agander T. K., Pakkenberg B. (2013). Screening of toll-like receptors expression in multiple system atrophy brains. *Neurochemical Research*.

[18] Pfaffl M. W. (2001). A new mathematical model for relative quantification in real-time RT-PCR. *Nucleic Acids Research*.

[19] Chen B.-S., Roche K. W. (2007). Regulation of NMDA receptors by phosphorylation. *Neuropharmacology*.

[20] Tingley W. G., Ehlers M. D., Kameyama K. (1997). Characterization of protein kinase A and protein kinase C phosphorylation of the N-methyl-D-aspartate receptor NR1 subunit using phosphorylation site-specific antibodies. *Journal of Biological Chemistry*.

[21] Scott D. B., Blanpied T. A., Swanson G. T., Zhang C., Ehlers M. D. (2001). An NMDA receptor ER retention signal regulated by phosphorylation and alternative splicing. *The Journal of Neuroscience*.

[22] Shaulsky G., Fuller D., Loomis W. F. (1998). A cAMP-phosphodiesterase controls PKA-dependent differentiation. *Development*.

[23] Sutton G., Chandler L. J. (2002). Activity-dependent NMDA receptor-mediated activation of protein kinase B/Akt in cortical neuronal cultures. *Journal of Neurochemistry*.

[24] Babiec W. E., Guglietta R., Jami S. A., Morishita W., Malenka R. C., O'Dell T. J. (2014). Ionotropic NMDA receptor signaling is required for the induction of long-term depression in the mouse hippocampal CA1 region. *Journal of Neuroscience*.

[25] Ko J., Zhang C., Arac D., Boucard A. A., Brunger A. T., Südhof T. C. (2009). Neuroligin-1 performs neurexin-dependent and neurexin-independent functions in synapse validation. *The EMBO Journal*.

[26] Budreck E. C., Kwon O.-B., Jung J. H. (2013). Neuroligin-1 controls synaptic abundance of NMDA-type glutamate receptors through extracellular coupling. *Proceedings of the National Academy of Sciences of the United States of America*.

[27] Voineskos A. N., Lett T. A. P., Lerch J. P. (2011). Neurexin-1 and frontal lobe white matter: an overlapping intermediate phenotype for schizophrenia and autism spectrum disorders. *PLoS ONE*.

[28] St Clair D., Blackwood D., Muir W. (1990). Association within a family of a balanced autosomal translocation with major mental illness. *The Lancet*.

[29] Millar J. K., Wilson-Annan J. C., Anderson S. (2000). Disruption of two novel genes by a translocation co-segregating with schizophrenia. *Human Molecular Genetics*.

[30] de Lacy N., King B. H. (2013). Revisiting the relationship between autism and schizophrenia: toward an integrated neurobiology. *Annual Review of Clinical Psychology*.

[31] Shepherd G. M. G. (2013). Corticostriatal connectivity and its role in disease. *Nature Reviews Neuroscience*.

[32] Hayashi-Takagi A., Araki Y., Nakamura M. (2014). PAKs inhibitors ameliorate schizophrenia-associated dendritic spine deterioration *in vitro* and *in vivo* during late adolescence. *Proceedings of the National Academy of Sciences of the United States of America*.

[33] Mandela P., Ma X.-M. (2012). Kalirin, a key player in synapse formation, is implicated in human diseases. *Neural Plasticity*.

